# Integrative machine learning and transcriptomic analysis identifies key molecular targets in MNPN-associated oral squamous cell carcinoma pathogenesis

**DOI:** 10.3389/fbinf.2025.1664576

**Published:** 2025-09-25

**Authors:** Xiangjun Wang, Panpan Jin, Juan Xu, Junyi Li, Mengzhen Ji

**Affiliations:** Department of Stomatology, The Third People’s Hospital of Henan Province, Zhengzhou, China

**Keywords:** oral squamous cell carcinoma (OSCC), betel nut nitrosamine, 3-(methylnitrosamino)propionitrile (MNPN), transcriptomic analysis, machine learning

## Abstract

**Background:**

Oral squamous cell carcinoma (OSCC) represents a significant global health challenge, with betel nut consumption being a major risk factor. 3-(methylnitrosamino)propionitrile (MNPN), a betel nut-derived nitrosamine, has been identified as a potential carcinogen, but its molecular targets in OSCC pathogenesis remain poorly understood.

**Methods:**

We employed a comprehensive computational framework integrating target prediction, transcriptomic analysis, weighted gene co-expression network analysis (WGCNA), and machine learning approaches. Four OSCC datasets from Gene Expression Omnibus (GEO) were analyzed, and MNPN targets were predicted using ChEMBL, PharmMapper, and SwissTargetPrediction databases. Machine learning algorithms (n = 127 combinations) were evaluated for optimal biomarker identification, with model interpretability assessed using SHAP (SHapley Additive exPlanations) analysis.

**Results:**

Target prediction identified 881 potential MNPN targets across three databases. WGCNA revealed 534 OSCC-associated differentially expressed genes, with 38 overlapping MNPN targets. Machine learning optimization identified 13 hub genes, with PLAU demonstrating the highest predictive performance (AUC = 0.944). SHAP analysis confirmed PLAU and PLOD3 as the most influential contributors to disease prediction. Functional enrichment analysis revealed MNPN targets’ involvement in xenobiotic response, hypoxic conditions, and aberrant tissue remodeling.

**Conclusion:**

This study provides the first comprehensive molecular characterization of MNPN-associated OSCC pathogenesis, identifying PLAU as a critical therapeutic target with exceptional diagnostic potential. Our findings establish a foundation for developing targeted interventions for betel nut nitrosamine-associated oral cancers and demonstrate the power of integrative computational approaches in environmental carcinogen research.

## 1 Introduction

OSCC represents the most prevalent malignancy of the oral cavity, accounting for approximately 90% of all oral cancers and constituting a significant global health challenge. With an estimated annual incidence exceeding 350,000 cases worldwide, OSCC ranks among the ten most common cancers globally, exhibiting particularly high prevalence rates in South and Southeast Asian populations (Tan et al., 2023; Prokopczyk et al., 1987). The disease is characterized by aggressive local invasion, high propensity for lymph node metastasis, and substantial morbidity due to its impact on essential functions including speech, swallowing, and facial aesthetics. Despite advances in multimodal treatment approaches encompassing surgery, radiotherapy, and chemotherapy, the 5-year survival rate for OSCC remains disappointingly low at approximately 50%–60% (Ng et al., 2017; Fatima et al., 2024), primarily attributed to late-stage diagnosis and limited understanding of molecular mechanisms underlying disease progression. The heterogeneous nature of OSCC, combined with its complex etiology involving multiple risk factors, necessitates comprehensive molecular characterization to identify novel therapeutic targets and develop precision medicine approaches.

Betel nut (Areca catechu) consumption represents one of the most significant and well-established risk factors for OSCC development, particularly in regions where this practice is culturally embedded, including India, Taiwan region, and other Asian-Pacific regions (Li et al., 2019). The International Agency for Research on Cancer (IARC) has classified betel nut as a Group 1 carcinogen. Studies indicate that the oral carcinogenic effects induced by betel nut are attributed to arecoline, reactive oxygen species, and nitrosamines (Warnakulasuriya and Chen, 2022). In betel nut, arecoline constitutes the primary alkaloid component at concentrations ranging from 0.1% to 0.7% of dry weight, followed by guvacine (0.19%–0.72%), arecaidine (0.31%–0.66%), and guvacoline (0.03%–0.06%) in fresh seeds (Gupta et al., 2020). These alkaloids contribute approximately 0.15%–0.70% of the total betel nut composition and have been extensively studied for their genotoxic and cytotoxic properties. Furthermore, betel nut consumption involves complex metabolic processes. During this process, various nitrogen-containing compounds undergo chemical transformations in the presence of saliva, bacterial enzymes, and added lime (calcium hydroxide), leading to the formation of N-nitroso compounds. It is known that alkaloids undergo nitrosation in the oral cavity in the presence of nitrites and thiocyanates (Jeng et al., 2001). Currently, nitrosamine derivatives may play important roles in OSCC pathogenesis, but research remains insufficient; therefore, this study focuses on investigating a specific secondary metabolite.

N-nitroso compounds, commonly referred to as nitrosamines, constitute a diverse class of chemical carcinogens formed through nitrosation of secondary amines. These compounds exhibit distinct carcinogenic potencies and target organ specificities, with their carcinogenic potential stemming from metabolic activation to highly reactive alkylating agents that form DNA adducts, particularly at guanine residues, leading to mutagenic lesions and subsequent malignant transformation (Li and Hecht, 2022). In betel quid consumption, nitrosamine exposure involves multiple compound categories. When betel quid is consumed with tobacco, tobacco-specific nitrosamines are formed, including N-nitrosonornicotine (NNN) and 4-(methylnitrosamino)-1-(3-pyridyl)-1-butanone (NNK), both classified as Group 1 human carcinogens by IARC (Peterson et al., 2024). Additionally, other nitrosamines such as N-nitrosodimethylamine (NDMA), a Group 2A carcinogen, may be present due to environmental exposure and endogenous formation. Furthermore, betel quid-specific nitrosamines are formed through direct nitrosation of areca nut alkaloids, primarily arecoline, resulting in four major compounds: N-nitrosoguvacoline (NGL), 3-(methylnitrosamino)propionaldehyde (MNPA), N-nitrosoguvacine (NGC), and 3-(methylnitrosamino)propionitrile (MNPN). While NGL, MNPA, and NGC are classified as Group 3 by IARC, MNPN stands out as a Group 2B carcinogen and has demonstrated potent carcinogenic effects in animal studies (Rangani et al., 2025). MNPN, distinguished by its nitrile group connected to a propyl chain bearing a methylnitrosamino moiety, has been consistently detected in betel quid extracts and oral cavity samples from habitual users (Prokopczyk et al., 1987). The formation of MNPN occurs readily under the alkaline conditions created by slaked lime addition during betel quid preparation. Given its specific association with betel quid consumption and demonstrated carcinogenic potential, MNPN represents a critical target for mechanistic investigation in betel quid-associated oral carcinogenesis and serves as the primary focus of this study.

Previous studies investigating carcinogen-disease relationships have primarily utilized *in vitro* cell culture experiments, animal models, and basic bioinformatics analyses to assess carcinogenic potential and elucidate molecular mechanisms. Recent advances have introduced network pharmacology, WGCNA, and machine learning approaches as powerful tools for understanding complex disease pathogenesis and identifying biomarkers (Al-Tashi et al., 2023; Rafique et al., 2021). The present study employs a comprehensive analytical framework that integrates large-scale transcriptomic data mining from GEO datasets, WGCNA for co-expression module identification, network toxicology for target prediction, an extensive evaluation of 127 machine learning algorithm combinations for optimal biomarker selection, and SHAP analysis for model interpretation to elucidate the molecular mechanisms underlying MNPN-associated OSCC pathogenesis (Ponce Bobadilla et al., 2024).

## 2 Materials and methods

### 2.1 Data acquisition and preprocessing

Four human OSCC transcriptomic datasets (GSE30784, GSE37991, GSE25099, and GSE146483) (Jiang et al., 2022) were selected from the GEO database (RRID:SCR_005012). Datasets focused solely on tongue carcinoma or broader head and neck squamous cell carcinoma were excluded, as well as datasets with unconventional storage formats or processing issues. GSE30784 (n = 212) and GSE37991 (n = 80) served as the discovery cohort, while GSE25099 (n = 79) and GSE146483 (n = 11) comprised the validation cohort. We evaluated datasets based on both sample size and research relevance. We selected the larger-sample-size GSE30784 and the more research-relevant GSE37991, which contains OSCC data from male patients who regularly consume alcohol, chew areca nut, and smoke, as the training set to enhance the reliability of target identification, while the remaining smaller-sample-size datasets served as the testing set to validate the accuracy of the trained model. Raw expression data were processed using R software (version 4.4.2; RRID:SCR_001905). Quality control included probe annotation, removal of non-specific probes, log2 transformation, and quantile normalization using the limma package (version 3.62.2; RRID:SCR_010943). To mitigate batch effects, Surrogate Variable Analysis (SVA) was performed using the sva package (version 3.54.0; RRID:SCR_012836). Post-correction principal component analysis confirmed successful data harmonization.

### 2.2 MNPN target prediction

MNPN targets were predicted using three complementary databases: ChEMBL (https://www.ebi.ac.uk/chembl/; RRID:SCR_014042), SwissTargetPrediction (http://swisstargetprediction.ch/; RRID:SCR_023756), and PharmMapper (https://lilab-ecust.cn/pharmmapper/index.html; RRID:SCR_022604). ChEMBL employs structure-activity relationship analysis based on experimental bioactivity data from literature, depositing bioassay data and focusing on compounds with validated biological activities (Mendez et al., 2019). SwissTargetPrediction utilizes ligand-based similarity searching and updated data features for efficient prediction of protein targets of small molecules (Daina et al., 2019) PharmMapper identifies potential drug targets through large-scale reverse pharmacophore mapping using a comprehensive target pharmacophore database (Wang et al., 2017). The canonical SMILES notation (CN(CCC#N)N=O) was retrieved from PubChem (https://pubchem.ncbi.nlm.nih.gov/; RRID:SCR_004284). All predicted targets were filtered to retain only human proteins and mapped to official gene symbols using the org.Hs.eg.db package (version 3.20.0).

### 2.3 Differential gene expression analysis

Differential expression analysis was conducted using the limma package with empirical Bayes moderation. In the training set, normal individuals without OSCC or normal tissues from OSCC patients served as the control group (n = 85), while tumor tissues from OSCC patients comprised the experimental group (n = 207). DEGs were identified using false discovery rate (FDR)-adjusted p-value <0.05 and absolute log2 fold change >0.585 (1.5-fold change). Multiple testing correction was performed using the Benjamini-Hochberg method. Results were visualized through volcano plots using ggplot2 package (version 3.5.2; RRID:SCR_014601), with the top 5 most significant genes labeled.

### 2.4 Weighted gene co-expression network analysis

Scale-free co-expression networks were constructed using the WGCNA package (Langfelder and Horvath, 2008) (version 1.73; RRID:SCR_003302). Sample quality control included outlier removal by hierarchical clustering. Optimal soft thresholding power was determined by analyzing scale-free topology fit index (*R* ^2^ ≥ 0.8). Gene modules were identified through hierarchical clustering of the topological overlap matrix using dynamic tree-cutting with minimum module size = 50. Module-trait associations were evaluated using Pearson correlation analysis (|r| > 0.5, p < 0.05). Hub genes were identified based on intramodular connectivity and gene significance.

### 2.5 MNPN-associated target identification

MNPN-associated disease targets were identified through intersection analysis between predicted MNPN targets, differentially expressed genes, and hub genes from trait-associated WGCNA modules. Venn diagrams were generated using the ggvenn package (version 0.1.10) to visualize overlapping gene sets.

### 2.6 Functional enrichment analysis

Gene Ontology (GO; RRID:SCR_002811) and Kyoto Encyclopedia of Genes and Genomes (KEGG; RRID:SCR_012773) pathway enrichment analyses were performed using the clusterProfiler package (Wu et al., 2021) (version 4.14.4; RRID:SCR_016884). Enrichment significance was assessed using hypergeometric testing with FDR correction (adjusted p-value <0.05). Protein-protein interaction (PPI) networks were constructed using the STRING database (https://string-db.org/; version 12.0; RRID:SCR_005223) with confidence score threshold 0.4 and visualized using Cytoscape software (version 3.10.3; RRID:SCR_003032).

### 2.7 Machine learning-based biomarker discovery

A comprehensive machine learning framework evaluated twelve algorithms: Lasso regression, Ridge regression, Elastic Net (α = 0.1–0.9), Random Forest (RF), Gradient Boosting Machine (GBM), XGBoost, Linear Discriminant Analysis (LDA), Naive Bayes, stepwise GLM (forward/backward/both), glmBoost, plsRglm, and Support Vector Machine (SVM). All algorithms were implemented using the caret package (version 7.0.1) with 10-fold cross-validation repeated 5 times, generating 127 unique algorithm combinations. Models were trained on the discovery cohort and validated on the independent validation cohort. Performance was assessed using area under the ROC curve (AUC), sensitivity, specificity, and balanced accuracy. High-performance models (AUC >0.9) were subjected to ensemble learning using stacking methodology. A nomogram was constructed based on logistic regression algorithm designed for the binary classification outcome (OSCC *versus* Normal) for clinical risk assessment. Decision curve analysis (DCA) was performed to evaluate the clinical net benefit of the predictive models.

### 2.8 Model interpretability analysis

Model interpretability analysis was performed using 14 algorithms, including the 12 machine learning algorithms and K-Nearest Neighbors (KNN) as well as ensemble algorithm glmBoost + LDA to quantify individual feature contributions to prediction outcomes. SHAP values were computed using the kernelshap package (version 0.7.0) and visualized with the shapviz (version 0.10.1) package. The analysis included global feature importance assessment through mean absolute SHAP values, feature interactions via dependence plots, and individual prediction explanations through force plots for representative samples. Additional model interpretation was conducted using the DALEX package (version 2.4.3) to provide comprehensive explanations of model predictions and feature relationships.

## 3 Results

### 3.1 Identification of MNPN target proteins

**FIGURE 1:**
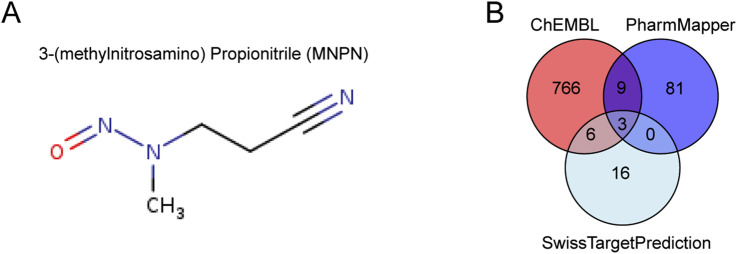
Molecular structure and target prediction analysis of MNPN. **(A)** Chemical structure of MNPN. **(B)** Venn diagram of predicted molecular targets from three databases: ChEMBL, PharmMapper, and SwissTargetPrediction.

To elucidate the potential biological targets of MNPN, we performed computational target prediction using three complementary databases: ChEMBL, PharmMapper, and SwissTargetPrediction. The chemical structure of MNPN is characterized by a nitrile group connected to a propyl chain bearing a methylnitrosamino moiety (Figure 1A). Target prediction analysis revealed distinct sets of potential molecular targets across the three databases (Figure 1B). ChEMBL identified 784 potential targets, PharmMapper predicted 93 targets, and SwissTargetPrediction suggested 25 targets (Supplementary Tables S1–S3). The Venn diagram analysis demonstrated the complementary nature of different prediction algorithms, with each database contributing unique target predictions alongside shared targets. To comprehensively capture the potential biological activities of MNPN, we selected the union of all predicted targets from the three databases for subsequent downstream analysis.

### 3.2 Transcriptomic data processing and co-expression network analysis

**FIGURE 2:**
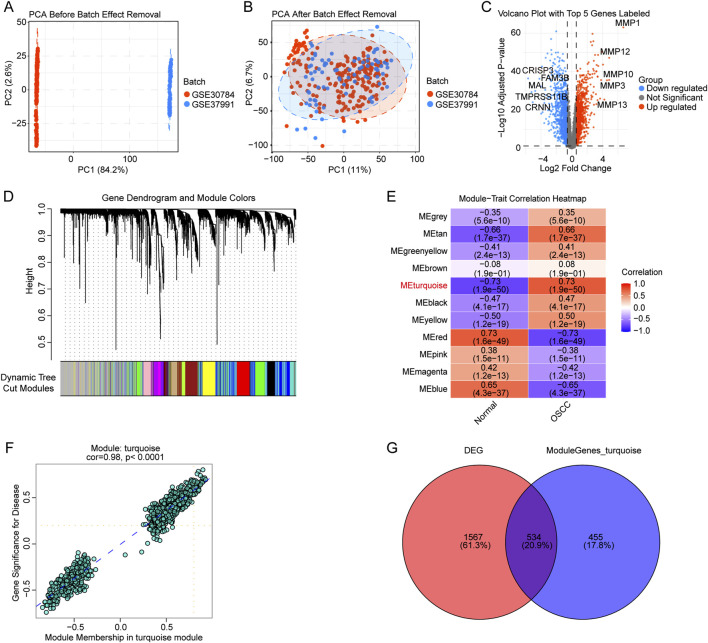
Transcriptomic analysis workflow and identification of key gene modules. **(A)** PCA plot before batch effect removal. **(B)** PCA plot after batch effect removal. **(C)** Volcano plot of DEGs with the top 5 genes labeled. Red and blue dots represent significantly up- and downregulated genes. **(D)** Gene dendrogram from WGCNA with color-coded modules below. **(E)** Module-trait correlation heatmap showing the association between gene modules and clinical traits. Correlation coefficients and p-values are displayed, with red indicating positive correlation and blue indicating negative correlation. **(F)** Scatter plot of gene significance *versus* module membership for the turquoise module (correlation = 0.98; p < 0.0001). **(G)** Venn diagram showing the overlap between DEGs and genes in the turquoise module.

To identify key gene modules associated with OSCC, we performed comprehensive transcriptomic analysis using two publicly available datasets (GSE23991 and GSE37991). Initial principal component analysis revealed distinct clustering of samples by dataset, indicating the presence of batch effects (Figure 2A). After applying batch effect correction using normalization, the principal component analysis (PCA) plot demonstrated improved sample distribution with reduced technical variation while preserving biological differences (Figure 2B).

Differential expression analysis comparing the OSCC group with the normal control group identified 2,101 significantly dysregulated genes, with 1,079 upregulated and 1,022 downregulated genes (Figure 2C; Supplementary Table S4). The volcano plot highlights the top 5 most significantly altered genes.

To explore co-expression patterns and identify functionally related gene modules, we conducted WGCNA. The gene dendrogram revealed distinct co-expression modules, each assigned a unique color identifier (Figure 2D; Supplementary Table S5). Module-trait correlation analysis demonstrated varying associations between gene modules and clinical traits, with several modules showing significant correlations (Figure 2E). Notably, the turquoise module exhibited strong correlation with OSCC (correlation coefficient = 0.73, p = 1.9e-50).

Further analysis of the turquoise module revealed a high correlation between gene significance and module membership (r = 0.98), indicating that genes central to this module are also highly associated with the trait of interest (Figure 2F). To identify the most relevant genes for downstream analysis, we examined the intersection between differentially expressed genes and the turquoise module. This analysis revealed 534 overlapping genes, representing high-confidence candidates that are both differentially expressed and co-regulated in a trait-associated network (Figure 2G).

### 3.3 Identification of MNPN-associated disease targets in OSCC

**FIGURE 3:**
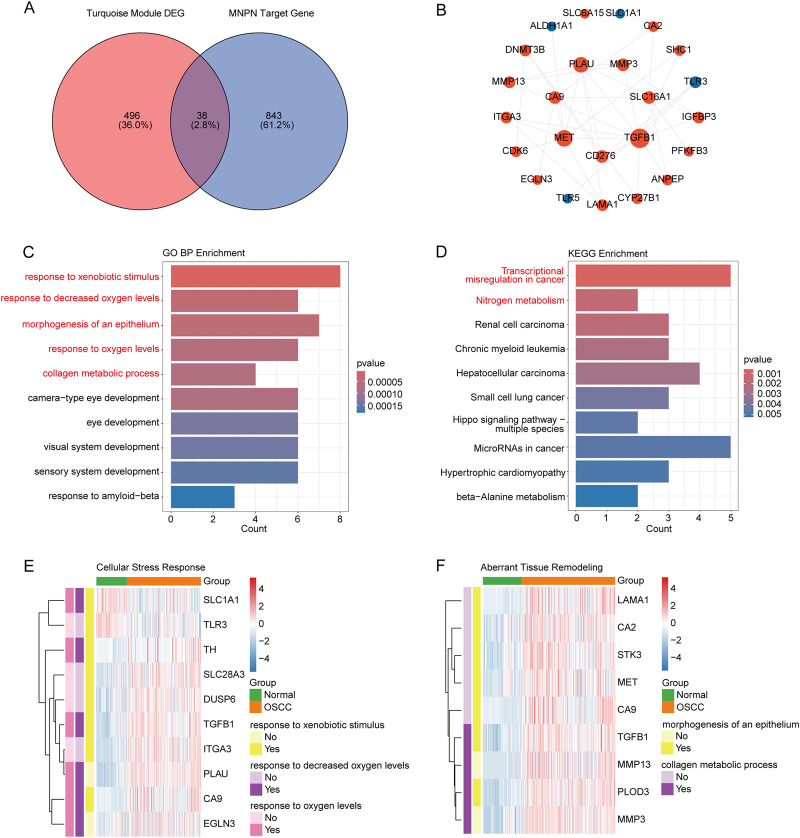
Identification and functional analysis of MNPN-associated targets in OSCC. **(A)** Venn diagram showing the intersection of MNPN target genes predicted by databases with OSCC-related differential genes from the turquoise module. **(B)** PPI network of the intersected gene-encoded proteins constructed using STRING database. Node colors: red indicates upregulated DEGs, blue indicates downregulated DEGs. Node size represents the degree of connectivity. **(C)** GO biological process enrichment analysis of the 38 target genes. Color gradients in panels represent statistical significance levels (p-values). **(D)** KEGG pathway enrichment analysis of the 38 target genes. **(E)** Heatmap showing expression patterns of genes involved in cellular stress response pathways. **(F)** Heatmap showing expression patterns of genes associated with aberrant tissue remodeling pathways.

To elucidate the molecular mechanisms underlying MNPN’s pathogenic effects in OSCC, we performed target prediction and intersection analysis. The intersection analysis between MNPN target proteins predicted from three independent databases (ChEMBL, PharmMapper, and SwissTargetPrediction) and OSCC-related differential genes from the turquoise module identified 38 potential key targets involved in MNPN-mediated oncogenic effects (Figure 3A; Supplementary Table S6).

The PPI network analysis revealed complex interconnections among the target genes, with only connected nodes displayed in the network (Figure 3B; Supplementary Table S7). Isolated nodes without protein-protein interactions were excluded from visualization. In PPI network topology analysis, proteins with high degree centrality typically serve as key regulatory factors, while betweenness centrality reflects the bridging role of proteins, and clustering coefficient indicates local network density. From our network analysis, nodes such as TGFB1, MET, and PLAU demonstrated high connectivity, suggesting these proteins function as critical regulatory factors in MNPN’s pathogenic mechanism.

Functional characterization through GO and KEGG enrichment analyses of the 38 MNPN-associated target genes revealed comprehensive molecular insights into MNPN’s oncogenic action mechanisms. GO biological process analysis demonstrated significant enrichment in processes including response to xenobiotic stimuli, response to decreased oxygen levels, morphogenesis of epithelium, collagen metabolic processes, and various developmental processes (Figure 3C; Supplementary Table S8). These significant pathways indicate that MNPN may promote OSCC progression primarily through dysregulation of cellular stress responses and aberrant tissue remodeling. KEGG pathway analysis highlighted enrichment in cancer-promoting pathways, including transcriptional misregulation in cancer and nitrogen metabolism (Figure 3D; Supplementary Table S9). These KEGG pathways indicate that MNPN may promote OSCC progression through activation of oncogenic signaling cascades and metabolic reprogramming.

However, GO pathways showed more significant enrichment than KEGG pathways, suggesting that GO-enriched pathways may be more closely associated with OSCC pathogenesis. We specifically highlight the key pathways and genes involved in cellular stress response and aberrant tissue remodeling through heatmap analysis (Figures 3E,F). The cellular stress response heatmap reveals that genes such as PLAU, CA9, and TGFB1 are prominently involved in xenobiotic response and hypoxic conditions, while the tissue remodeling heatmap demonstrates that PLOD3 and TGFB1 play critical roles in collagen metabolism and epithelial morphogenesis. The multi-pathway involvement suggests MNPN’s complex role as a multi-target oncogenic factor contributing to malignant transformation and tumor maintenance in OSCC development.

### 3.4 Machine learning-based construction of predictive model and identification of hub genes with clinical utility assessment

To identify the most predictive MNPN-associated genes for OSCC diagnosis, we employed multiple machine learning algorithms on the 38 MNPN-OSCC related differential gene targets identified. We evaluated the performance of various algorithms using both training and testing datasets to optimize both predictive accuracy and gene number.

**FIGURE 4:**
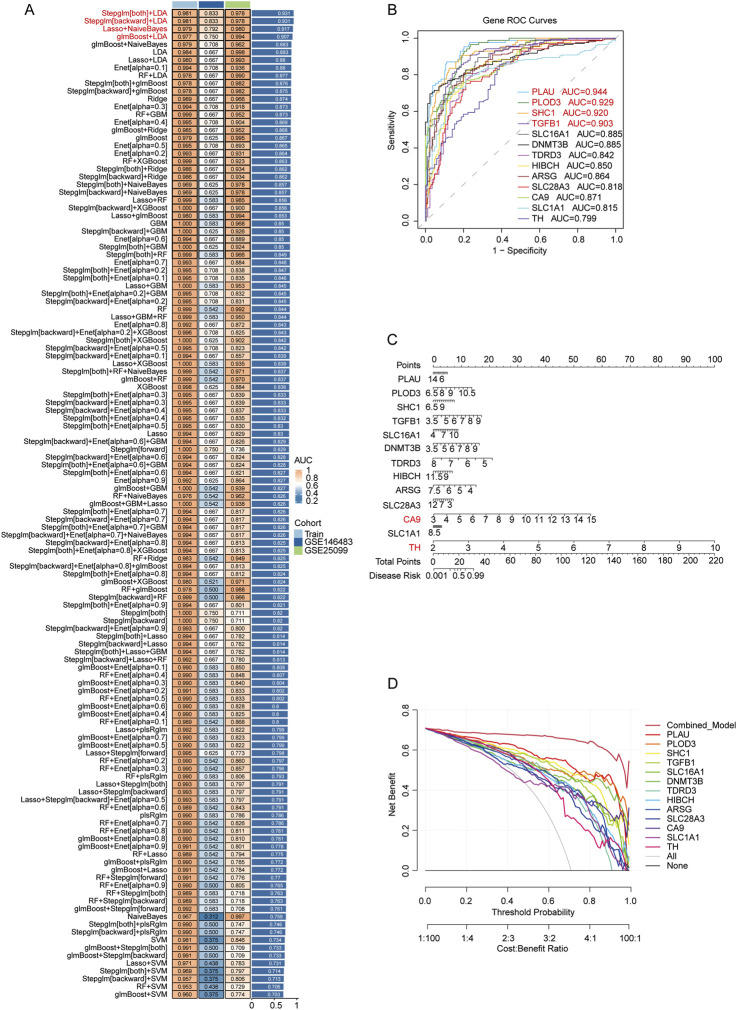
Machine learning-based construction of predictive model and identification of hub genes with clinical utility assessment. **(A)** Performance comparison of different machine learning algorithms showing AUC values in training and testing datasets. **(B)** ROC curves of the 13 hub genes identified by the selected glmBoost + LDA algorithm. **(C)** Nomogram constructed based on the 13 hub genes using logistic regression algorithm for risk prediction. **(D)** DCA of the logistic regression algorithm showing the relationship between clinical net benefit and threshold probability for different gene combinations.

Comprehensive comparison of various machine learning approaches revealed distinct performance patterns across different algorithms (Figure 4A; Supplementary Table S10). Considering the dual criteria of relatively high AUC values and a gene signature size of approximately 10 genes, the glmBoost + LDA algorithm exhibited satisfactory performance and was selected as the predictive framework, identifying 13 hub genes from the training dataset (Supplementary Table S11).

The ROC analysis of the 13 hub genes selected by the glmBoost + LDA algorithm showed excellent discriminatory power, with AUC values ranging from 0.799 to 0.944 (Figure 4B). Notably, PLAU, PLOD3, SHC1 and TGFB1 exhibited the highest predictive accuracy with AUC values exceeding 0.9, indicating their strong potential as diagnostic biomarkers for OSCC.

The nomogram visualization revealed the clinical risk prediction value of these hub genes for OSCC (Figure 4C). The nomogram converts gene expression levels into risk scores, where genes with larger score ranges have greater impact on OSCC risk assessment, particularly CA9 and TH in our analysis. DCA evaluated the clinical net benefit of different genes across various risk thresholds (Figure 4D). In the decision curve, “None” represents no treatment with zero net benefit, while “All” represents treating all patients without discrimination. All single-gene models demonstrated higher net benefit compared to the “All” strategy, with PLAU showing the highest clinical net benefit among single-gene models across most threshold ranges. Furthermore, among all models, “Combined_Model” demonstrated optimal performance, indicating that the multi-gene combined model provides superior clinical net benefit for OSCC diagnosis. This approach refined the 38 MNPN-related targets into 13 highly predictive hub genes critical for MNPN-driven OSCC pathogenesis, demonstrating their predictive performance, clinical risk assessment value, and clinical benefit potential.

### 3.5 SHAP analysis identifies key hub genes contributing to disease prediction

**FIGURE 5:**
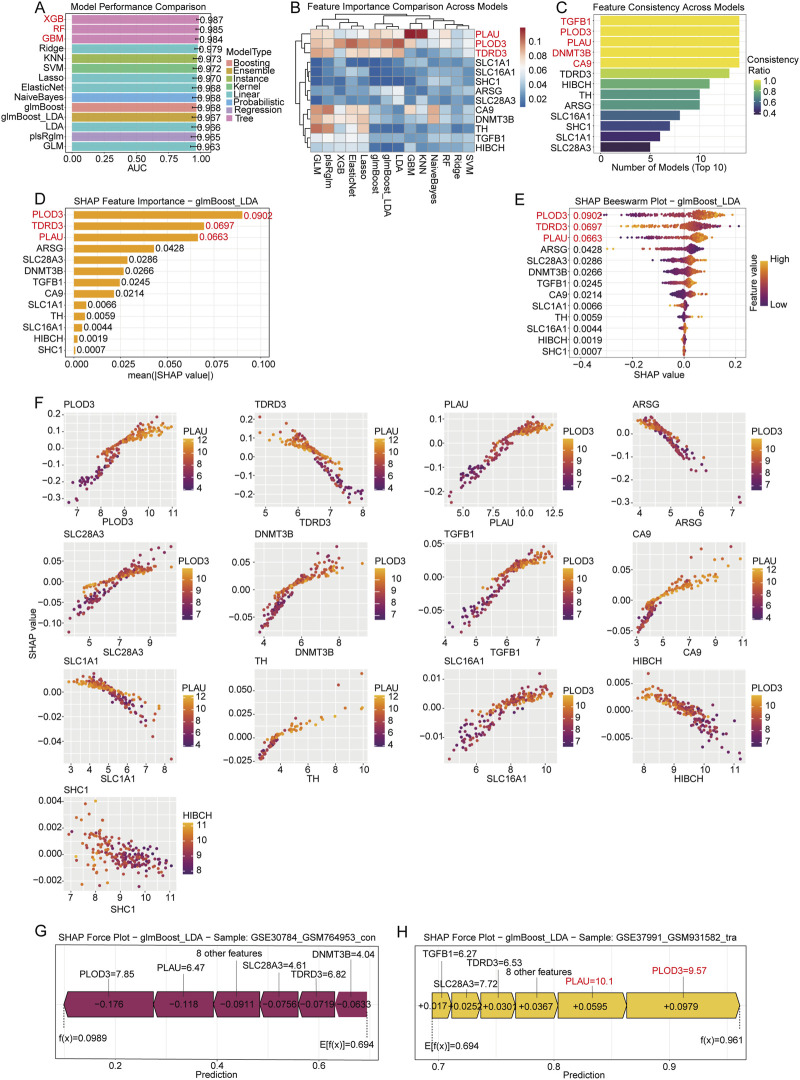
SHAP analysis identifies key hub genes contributing to disease prediction. **(A)** Model performance comparison showing AUC values for different machine learning algorithms. **(B)** Heatmap displaying feature importance scores for hub genes across different algorithms. **(C)** Bar plot showing consistency analysis of gene ranking across multiple algorithms. **(D)** Mean absolute SHAP values bar plot for each of the 13 hub genes in the glmBoost + LDA algorithm. **(E)** SHAP value distribution for each gene displayed as beeswarm plots in the glmBoost + LDA algorithm. Color scale represents expression levels of genes. **(F)** SHAP dependence plots showing the relationship between gene expression levels and SHAP values for individual hub genes in the glmBoost + LDA algorithm. **(G)** SHAP force plot for a randomly selected control sample (GSE30784_GSM764953_con), showing individual gene contributions to the final prediction. **(H)** SHAP force plot for a randomly selected disease sample (GSE37991_GSM931582_tra), showing individual gene contributes to the disease prediction.

To further elucidate the relative importance of the 13 hub genes in disease classification, we employed SHAP analysis to provide interpretable insights into our machine learning models. Model performance evaluation demonstrated varying predictive capability across 14 algorithms (Figure 5A). Among these algorithms, tree-based methods including XGBoost, RF, and GBM achieved the highest performance (AUC >0.98). The remaining 11 algorithms also demonstrated excellent predictive capability, with AUC values all exceeding 0.96. The consistently high performance across diverse algorithmic categories validated the robustness of our hub gene signature and made these models particularly suitable for subsequent SHAP interpretation analysis.

The feature importance heatmap across different models revealed varying contributions of hub genes to disease prediction (Figure 5B; Supplementary Table S12). PLAU, PLOD3, and TDRD3 demonstrated high importance scores across the majority of algorithms. The consistency analysis across algorithms (Figure 5C) showed that TGFB1, PLOD3, PLAU, DNMT3B, and CA9 exhibited high consistency across different algorithms. Collectively, PLAU and PLOD3 emerged as the consistently important genes for OSCC prediction across most of the evaluated algorithms.

The SHAP analysis using the glmBoost + LDA algorithm revealed differential contributions of hub genes to disease prediction (Figures 5D,E). Among these genes, PLAU, TDRD3, and PLOD3 were the most influential genes, exhibiting the highest mean absolute SHAP values. The beeswarm plots demonstrated that high expression points (yellow) with positive SHAP values indicate increased likelihood of OSCC classification, promoting disease development, while negative values suggest a protective role against disease progression. This analysis clearly indicated that PLOD3 and PLAU drive OSCC development.

SHAP dependence plots (Figure 5F) illustrated the complex relationships between gene expression levels and their predictive contributions. For instance, PLOD3 showed a positive correlation between expression levels and SHAP values, indicating that higher expression consistently increased disease prediction probability. Conversely, TDRD3 exhibited a negative correlation between expression levels and SHAP values, suggesting that higher expression reduced disease prediction probability. Their contributions varied based on expression thresholds and potential gene-gene interactions.

Individual sample analysis through SHAP force plots provided mechanistic insights into model predictions (Figures 5G,H). In randomly selected control samples, most hub genes contributed negatively to disease probability, maintaining the prediction below the baseline. In contrast, disease samples showed predominant positive contributions from key genes such as PLAU and PLOD3, collectively driving the prediction toward disease classification. These results validate the biological relevance of our identified hub genes and demonstrate their potential as diagnostic biomarkers for OSCC.

## 4 Discussion

This study presents a comprehensive computational framework integrating toxicology, transcriptomic analysis, machine learning approaches, and SHAP analysis to elucidate the molecular mechanisms underlying MNPN-mediated oral squamous cell carcinoma pathogenesis. Our findings provide novel insights into the toxicological profile of this betel nut-derived nitrosamine and identify critical therapeutic targets for OSCC prevention and treatment.

### 4.1 MNPN as a critical carcinogenic component in betel nut-associated OSCC

Our computational target prediction revealed that MNPN exhibits broad molecular promiscuity, with 881 predicted targets from ChEMBL, PharmMapper, and SwissTargetPrediction databases. WGCNA identified 534 DEGs highly correlated with OSCC, among which 38 were MNPN-related targets. These overlapping genes provide compelling evidence for the direct involvement of this nitrosamine in oral carcinogenesis.

The functional enrichment analysis revealed that MNPN-associated targets are significantly involved in response to xenobiotic stimuli, hypoxic conditions, epithelial morphogenesis, and collagen metabolism. These biological processes are fundamental to cancer initiation and progression, supporting the notion that MNPN contributes to OSCC through disruption of cellular homeostasis, tissue architecture, and stress response mechanisms. The dysregulation of cellular stress responses is particularly relevant, as it encompasses both the hypoxic stress response and xenobiotic detoxification pathways. This reflects the cellular attempt to cope with oxygen deprivation and detoxify MNPN, potentially leading to the formation of more reactive metabolites that cause DNA damage and mutagenesis. This is consistent with previous studies that betel nut-derived MNPN induces aberrant cell proliferation in OSCC (Chen et al., 2017).

### 4.2 PLAU as a critical nexus in MNPN-mediated OSCC pathogenesis

Among the 13 hub genes identified through machine learning optimization, PLAU (plasminogen activator, urokinase) emerged as the most significant contributor to OSCC prediction, exhibiting the highest AUC value and frequently ranking among the top contributors in SHAP analysis across multiple algorithms, indicating its stable contribution to disease prediction. PLAU encodes urokinase-type plasminogen activator (uPA), a serine protease that plays pivotal roles in extracellular matrix degradation, cell migration, invasion, and angiogenesis. The protein functions by converting plasminogen to plasmin, which subsequently degrades fibrin and various extracellular matrix components, facilitating tumor cell invasion and metastasis.

The identification of PLAU as a primary MNPN target with exceptional predictive power for OSCC provides several mechanistic insights. Firstly, PLAU upregulation in response to MNPN exposure may enhance the invasive capacity of oral epithelial cells, promoting malignant transformation and tumor progression. Recent studies have confirmed that PLAU promotes cell proliferation and epithelial-mesenchymal transition across multiple cancer types including head and neck squamous cell carcinoma (Chen et al., 2021), pancreatic ductal adenocarcinoma (Hosen et al., 2022), and triple-negative breast cancer (Sarno et al., 2022), with higher expression correlating with poorer clinical outcomes. Additionally, the SHAP analysis demonstrates the contribution of genes to disease prediction, revealing that PLAU’s positive correlation with disease probability is consistent across expression levels. Furthermore, PLAU expression is known to be induced by hypoxic conditions through HIF-1α activation (Chen et al., 2023a; Nishi et al., 2016), which aligns with our GO enrichment results showing MNPN targets’ involvement in hypoxic response pathways, including response to decreased oxygen levels, response to oxygen levels, and response to hypoxia. This connection suggests that PLAU may serve as a crucial mediator linking MNPN exposure to hypoxia-induced oncogenic signaling in oral tissues.

The clinical relevance of PLAU in OSCC is well-established, with numerous studies demonstrating its association with poor prognosis, increased metastatic potential, and treatment resistance (Bacchiocchi et al., 2008). Moreover, the single-gene model based on PLAU demonstrated the highest clinical net benefit in DCA, further supporting its clinical utility. Our findings extend this knowledge by providing the first evidence linking PLAU upregulation to specific environmental carcinogen exposure, particularly MNPN from betel nut consumption. This connection offers a molecular explanation for the aggressive nature of betel nut-associated oral cancers and suggests that PLAU could serve as both a biomarker for MNPN exposure and a therapeutic target for intervention.

### 4.3 Comparative analysis with previous research

Our computational approach reveals both consistencies and novel findings compared to previous investigations of betel nut carcinogenesis. Traditional studies have primarily focused on arecoline as the major carcinogenic component, with limited attention to nitrosamine derivatives like MNPN. While arecoline has been shown to induce cell proliferation, invasion and migration, genotoxicity, and inflammatory responses (Gocol et al., 2023), our study demonstrates that MNPN targets distinct molecular pathways that complement and potentially amplify arecoline’s carcinogenic effects.

Previous toxicological studies of betel nut components have identified several overlapping targets with our findings, including genes involved in cell cycle regulation, apoptosis, and inflammation. However, the specific identification of PLAU as a critical MNPN target represents a novel contribution to the field. Earlier proteomic and transcriptomic studies of OSCC have reported PLAU upregulation, but none have established its connection to specific betel nut-derived carcinogens.

The machine learning approach employed in this study, utilizing 127 algorithm combinations, represents a significant methodological advancement over previous biomarker identification studies that typically rely on single statistical methods. Our glmBoost + LDA algorithm achieved superior performance compared to conventional approaches, demonstrating the value of ensemble methods in identifying robust biomarker signatures.

### 4.4 Additional hub genes and therapeutic implications

Beyond PLAU, our analysis identified 12 additional hub genes that warrant further investigation as potential therapeutic targets. Among these, PLOD3 (procollagen-lysine, 2-oxoglutarate 5-dioxygenase 3) showed the second-highest predictive performance and represents another critical component in extracellular matrix remodeling. PLOD3 is essential for collagen hydroxylation and cross-linking, processes that are frequently dysregulated in cancer-associated fibrosis and tumor stroma formation (Qi and Xu, 2018). SHC1 (SHC adaptor protein 1) and TGFB1 (transforming growth factor beta 1) also demonstrated excellent predictive performance (AUC >0.9), representing key nodes in growth factor signaling and cellular transformation pathways (Chen et al., 2022; Liu et al., 2021).

Other hub genes include CA9 (carbonic anhydrase 9), which plays crucial roles in pH regulation and hypoxic adaptation (Giatromanolaki et al., 2020); DNMT3B (DNA methyltransferase 3 beta), involved in epigenetic modifications and gene silencing (Heawchaiyaphum et al., 2021); and several solute carriers (SLC16A1, SLC1A1, SLC28A3) that regulate metabolic transport processes (Pizzagalli et al., 2021). HIBCH (3-hydroxyisobutyryl-CoA hydrolase) and ARSG (arylsulfatase G) contribute to metabolic pathways (Wang et al., 2021; Poterala-Hejmo et al., 2020), while TH (tyrosine hydroxylase) and TDRD3 (tudor domain containing 3) are involved in neurotransmitter synthesis and RNA processing, respectively (Yi et al., 2024; Chen et al., 2023b). The convergence of MNPN targeting on these fundamental signaling and metabolic molecules underscores the multi-faceted nature of nitrosamine-induced carcinogenesis.

### 4.5 Novel hypotheses

Our findings support a novel hypothesis that MNPN promotes OSCC through coordinated disruption of tissue homeostasis mechanisms. The simultaneous targeting of proteolytic enzymes (PLAU), matrix synthesis enzymes (PLOD3), growth factor signaling (TGFB1, SHC1), metabolic regulators (CA9, SLC family members), and epigenetic modifiers (DNMT3B) creates a cellular environment conducive to malignant transformation. This multi-target mechanism may explain why betel nut-associated cancers often exhibit poor treatment responses.

The enrichment of MNPN targets in hypoxic response pathways suggests an additional mechanism whereby this nitrosamine may sensitize oral tissues to hypoxic stress, a common feature of the oral microenvironment. This sensitization could accelerate the progression from premalignant lesions to invasive carcinoma, particularly in individuals with concurrent risk factors such as tobacco use or poor oral hygiene.

### 4.6 Clinical implications and translational potential

The identification of PLAU as a primary MNPN target with exceptional diagnostic accuracy has immediate clinical implications for OSCC screening and risk assessment. PLAU expression levels, either alone or in combination with other hub genes, could serve as biomarkers for early detection of betel nut-associated oral malignancies. This is particularly relevant for high-risk populations in endemic regions where routine screening could significantly impact disease outcomes.

Furthermore, the established role of PLAU in cancer invasion and metastasis makes it an attractive therapeutic target. Several PLAU inhibitors, including small molecules and monoclonal antibodies, are currently in preclinical and clinical development for various cancer types (Zhai et al., 2022). Our findings provide a strong rationale for evaluating these agents specifically in betel nut-associated OSCC, potentially leading to targeted prevention or treatment strategies.

The machine learning framework developed in this study also has broader applications for environmental carcinogen research. The integration of target prediction, transcriptomic analysis, and interpretable machine learning could be applied to investigate other carcinogen-disease relationships, accelerating the identification of novel therapeutic targets and biomarkers.

### 4.7 Study limitations and future directions

Several limitations of this study should be acknowledged. Our analysis relies entirely on computational predictions and public database mining, lacking experimental validation of the proposed MNPN-PLAU interaction. Future studies should employ molecular techniques such as surface plasmon resonance, molecular docking simulations with experimental validation, and cell-based assays to confirm direct binding and functional relationships. Additionally, the transcriptomic data used in this study were derived from mixed OSCC populations that may not specifically represent betel nut-associated cases. Ideally, future investigations should focus on transcriptomic profiles from OSCC patients with confirmed betel nut exposure history to enhance the specificity of our findings. Moreover, the cross-sectional nature of the available datasets limits our ability to assess temporal relationships between MNPN exposure, gene expression changes, and disease progression. Longitudinal studies tracking individuals from initial betel nut exposure through premalignant changes to invasive carcinoma would provide crucial insights into the temporal dynamics of MNPN-mediated carcinogenesis.

Future research directions should include experimental validation of MNPN-PLAU interactions using biochemical and cellular assays, development of MNPN-specific exposure biomarkers for epidemiological studies, investigation of genetic polymorphisms in PLAU and other hub genes that may modify individual susceptibility to MNPN-induced carcinogenesis, evaluation of PLAU inhibitors as chemopreventive agents in high-risk populations, and expansion of the analytical framework to investigate other betel nut-derived nitrosamines and their molecular targets.

## 5 Conclusion

This study provides the first comprehensive molecular characterization of MNPN-associated OSCC pathogenesis, identifying PLAU as a critical therapeutic target with exceptional diagnostic and prognostic potential. Our findings represent a paradigm shift from traditional focus on arecoline to secondary metabolite nitrosamines and establish a foundation for developing targeted interventions for this global health challenge. The integration of computational toxicology, machine learning, and SHAP approaches demonstrates the power of systems-level analysis in elucidating complex environmental carcinogen mechanisms and identifying novel therapeutic opportunities.

## Data Availability

The original contributions presented in the study are included in the article/Supplementary Material, further inquiries can be directed to the corresponding author.
